# The Re-Emergence of Rift Valley Fever in Mananjary District, Madagascar in 2021: A Call for Action

**DOI:** 10.3390/pathogens13030257

**Published:** 2024-03-16

**Authors:** Aina Nirina Harimanana, Soa Fy Andriamandimby, Dany Bakoly Ranoaritiana, Laurence Randrianasolo, Judickaelle Irinantenaina, Nirina Nantenaina Ranoelison, Jean Théophile Rafisandrantatsoa, Miamina Fidy Ankasitrahana, Antso Hasina Raherinandrasana, Manuela Vololoniaina Andriamahatana, Michael Luciano Tantely, Romain Girod, Philippe Dussart, Vincent Lacoste, Rindra Vatosoa Randremanana

**Affiliations:** 1 Epidemiology and Clinical Research Unit, Institut Pasteur de Madagascar, Antananarivo 101, Madagascar; laurence@pasteur.mg (L.R.); judi@pasteur.mg (J.I.); nranoelison@pasteur.mg (N.N.R.); rrandrem@pasteur.mg (R.V.R.); 2 Virology Unit, Institut Pasteur de Madagascar, Antananarivo 101, Madagascar; soafy@pasteur.mg (S.F.A.); theo@pasteur.mg (J.T.R.); pdussart@pasteur.mg (P.D.); vlacoste@pasteur.mg (V.L.); 3 Direction de la Veille Sanitaire, de la Surveillance Epidémiologique et de la Riposte, Ministère de la Santé Publique, Antananarivo 101, Madagascar; rdanytia2@gmail.com (D.B.R.); miamina.fidy.ankasitrahana@umontreal.ca (M.F.A.); rantsohasina@yahoo.com (A.H.R.); manuelachristophere@gmail.com (M.V.A.); 4 Public Health Department, Faculty of Medicine, University of Antananarivo, Antananarivo 101, Madagascar; 5 Medical Entomology Unit, Institut Pasteur de Madagascar, Antananarivo 101, Madagascar; lucinambi@pasteur.mg (M.L.T.); rgirod@pasteur.mg (R.G.)

**Keywords:** cattle, disease outbreaks, humans, Madagascar, One Health approach, rift valley fever

## Abstract

An epizootic of rift valley fever (RVF) was suspected on 21 February 2021 in various districts of Madagascar, with a lab confirmation on 1 April 2021. A cross-sectional survey aiming to detect cases of RVF in humans and to study the circulation of rift valley fever virus (RVFV) in animals was conducted from 22 April to 4 May 2021 in the district of Mananjary. Blood samples from cattle and humans were tested using serological and molecular techniques. In cattle, the circulation of RVFV was confirmed between 5 February and 4 May 2021. The positivity rates of anti-RVFV IgG and IgM were 60% and 40%, respectively. In humans, the circulation of RVFV was observed from 1 April to 5 May 2021. The positivity rate of RVFV was estimated to be 11.7% by combining the results of the molecular and serological approaches. Of the 103 individuals who agreed to participate in the survey, 3 were determined to be positive by RT-PCR, and 10 had anti-RVFV IgM. Among them, one was positive for both. Given that previous studies have reported the circulation of RVFV during inter-epidemic periods and the occurrence of outbreaks due to imported RVFV in Madagascar, our findings suggest the importance of strengthening RVF surveillance from a “One Health” perspective by conducting syndromic and risk-based surveillance at the national and regional levels.

## 1. Introduction

Rift valley fever virus (RVFV) belongs to the *Phlebovirus* genus of the family *Phenuiviridae* (order Bunyavirales). This virus causes a zoonotic disease characterized by abortion storms and increased mortality rates in wild and domestic animals. Epizootic outbreaks of rift valley fever occur following periods of extensive rainfall and subsequent flooding. The disease can be transmitted to humans via infectious mosquito bites or through direct contact with products from infected animals. RVFV is one of the eight pathogens included in the Bluepoint list created by the World Health Organization (WHO) [1,2]. The WHO has raised awareness of the threat of arbovirus epidemics. In 2022, a global initiative for arboviral diseases was launched to prepare all nations for a possible arbovirus outbreak [3].

RVFV can be detected by RT-PCR in blood samples up to 4–5 days after the onset of symptoms. Antibody testing using enzyme-linked immuno-assays can detect an early response to a recent infection, showing the presence of RVFV-specific IgM antibodies for a brief period starting on days 5–6 after the onset of symptoms. Afterwards, RVFV-specific IgG antibodies appear and persist for years [4].

In Madagascar, RVFV was first isolated from mosquitoes in the Moramanga district, located between the capital and the East Coast, in 1979. A rift valley fever (RVF) epizootic in cattle with high incidences of abortions and human illness was then reported in 1990 in Fenoarivo Atsinanana on the East Coast [5,6]. Abortions and deaths in cattle and human cases were also reported in the central highlands in 1991 [5,7]. From 2008 to 2009, outbreaks of RVF in humans and animals were reported on the northern and southern coasts and in the central highlands. The circulation of RVFV was reported in 82.9% (92/111) of the districts through a nationwide serologic survey of professionals at risk of RVF [8].

The surveillance of RVF in animals and humans has been set up as part of the “SEGA One Health” program. Three surveillance systems are currently in use in Madagascar: the monitoring of health events by community health workers (CHWs), integrated disease surveillance and response by health units for human health managed by the Direction de la Veille Sanitaire de la Surveillance Epidémiologique et Riposte (DVSSE-R), and animal health surveillance by veterinarians managed by the Direction des Services Vétérinaires (DSV). CHWs collect data on health and health-related events on a daily basis. For reporting purposes, data are collected and analyzed on a weekly basis based on epidemiological weeks (epi weeks) using Monday as the first day of the week following the recommendation of the Ministry of Public Health (MoPH) of Madagascar. During epi week 7/2021 (15–21 February 2021), cases of bovine and ovine abortions were notified by CHWs in the fokontany (lowest administrative units in Madagascar) of Antananabo in Vohipeno district (region of Vatovavy Fitovinany located in the southeast area) and in the fokontany of Andranomangatsiaka in the district of Betioky Atsimo (region of Atsimo Andrefana, located in the southwest area), and suspected cases of RFV in animals were raised in the “bulletin mensuel de surveillance en santé publique” (n°31/2021, June 2021). During epi week 12/2021 (22–28 March 2021), a notification of 750 cases of abortion in cattle and sheep was sent to the DSV by three regions: Diana (located in the northern area), Atsimo Andrefana, and Vatovavy Fitovinany. Two of the twenty-two samples received at the Institut Pasteur de Madagascar (IPM) for RVFV testing were confirmed positive by RT-PCR on 1 April 2021 (epi week 13/2021). In epi week 17/2021 (26 April–2 May 2021), the DVSSER reported the occurrence of an epizootic of RVF in cattle, goats, and sheep in five regions (Atsimo Andrefana, Vatovavy Fitovinany, Diana, Atsimo Atsinanana, and Alaotra Mangoro in the middle-east area) spread across 14 health districts, with a total of 4514 sick cases in all species and 1040 deaths (mortality rate of 25%). The mortality rate was the highest in cattle [50% (938/1876)] compared to other species, such as sheep [12% (15/123)] and goats [4% (86/2155)]. The region of Vatovavy Fitovinany was the most affected (1611 cases and 899 deaths) (source: RVF report n°001 of 30 April 2021).

Following this epizootic, the surveillance of cases of hemorrhagic fever was increased, particularly in the concerned districts. On 15 April 2021 (epi week 15/2021), the head of the Antsenavolo health facility (Mananjary district, Vatovavy Fitovinany region) identified a suspected human case of RVF. The case presented with fever and digestive bleeding, with a history of ingestion of the liver of an ox suspected of RVF. No biological confirmation was carried out for this patient. As a result, a multidisciplinary investigation was carried out by central and local teams in the Mananjary district from 22 April to 26 May 2021 to confirm the circulation of RVF.

## 2. Materials and Methods

### 2.1. Ethics

This survey was realized at the request of the MoPH to confirm the circulation of RVF in humans and assist local and MoPH actions and interventions. Prior to the mission, authorizations were obtained from regional and district health officials. Participation in the study was voluntary. Participants completed and signed a consent form in Malagasy. For all individuals aged between 7 and 18 years, assent was obtained with the consent of an adult family member. For all individuals under the age of 7, an adult consented to their participation.

### 2.2. Study Design

A cross-sectional survey for case detection study in humans and cattle (outbreak investigation) was carried out from 26 April to 4 May 2021.

### 2.3. Study Site

The Mananjary district is located in the southeastern part of Madagascar. The district comprises 40 communes (the district is the second level and the commune is the third level of administrative units in Madagascar), the majority of which are located in rural areas. Agriculture is the main source of income in this region. The average annual temperature ranges from 15 °C to 32 °C, and the average rainfall is around 2500 mm [9].

The communes were selected by a multidisciplinary team consisting of representatives of the MoH, the district, health centers, and veterinarians. The study sites were the communes where suspected or confirmed cases had been reported as follows: in Antsenavolo (12,699 inhabitants), where a suspected human case had been reported; in Anosimparihy (10,626 inhabitants), where a bovine case had been confirmed by RT-PCR; and in Andonabe (9690 inhabitants) and Ambohimiarina II (9878 inhabitants), where animal cases had been suspected by veterinarians.

### 2.4. Case Definitions

Initially, active case detection was carried out to identify any suspected cases. The search for human cases suspected of RVF was carried out for as far back as January 2021, according to the following criteria: (1) any person with an onset of acute fever and a negative malaria test who presented to the health facility, (2) any person who had been in contact with livestock suspected of having RVF, and (3) any person who had presented with a symptom of RVF. Suspected livestock cases were identified with the help of veterinary assistants in the selected communes. The date of onset of the disease was not restricted for livestock.

A confirmed case was defined as any person or animal with a positive laboratory result by either quantitative reverse transcriptase polymerase chain reaction (qRT-PCR) or ELISA for IgM antibodies to RVFV.

### 2.5. Data Collection

Eligible patients were identified from the health facility register and visited at home. At the same time, the population was informed of the survey and on the symptoms of RVF by the head of the health facility and the mayor of the commune. If a person was thought to be a suspected case, they were invited to join one of the team members. Questions were administered orally in Malagasy by a qualified team member, and data were collected on paper. The questionnaire included socio-demographic information, symptoms, and date of onset of the disease.

For livestock, veterinarians helped the team to identify the owner of the livestock and the symptoms exhibited by the animals.

### 2.6. Laboratory Analysis

A blood sample (5 mL) was collected from each human and animal participant for molecular and serological testing. Both human and animal specimens were tested by qRT-PCR for RVFV RNA using Lab-on-wheels, which was specifically sent on the field for this purpose. All serological assays were performed by the National Reference Laboratory for Arboviruses at IPM as follows: Human specimens were tested by ELISA for anti-RVFV IgM (in-house ELISA). Animal specimens were tested by ELISA for anti-RVFV IgM antibodies directed against the nucleoprotein (ID Screen^®^ Rift Valley Fever IgM Capture, Innovative Diagnostic, Grabels, France) and for IgG (in-house ELISA).

### 2.7. Statistical Analyses

Questionnaire data were entered into REDCap v10.0.23 [10,11] and analyzed using STATA 17 (StataCorp LP, College Station, TX, USA). We conducted a descriptive analysis on the main symptoms and the proportion of confirmed cases in humans and livestock.

## 3. Results

### 3.1. Description of RVF in Cattle

During the survey, the only species reported by the community and veterinarians to be affected by RVF were cattle. Only cattle were included in the study. Forty blood samples from cattle were collected from 23 households in the four investigated communes. The cattle ranged in age from 5 months to 12 years. The sex ratio was 0.5 (13 males and 27 females).

A total of 26 cattle showed symptoms. The delay between the onset of symptoms and sample collection ranged from 1 to 83 days (from 5 February 5 to 4 May 2021, corresponding to epi weeks 5 to 18/2021). The main symptoms reported were anorexia in 57.7% of cases (15/26), ocular symptoms in 38.5% (10/26), and diarrhea in 26.9% (7/26). Four animals, aged between 10 and 12 years, had suffered a miscarriage. One of them died.

None of the samples were confirmed by qRT-PCR. The anti-RVFV IgG seroprevalence rate was 60% (24/40), and the anti-RVFV IgM rate was 40% (16/40) (Table 1).

### 3.2. Description of Results for RVF in Humans

Overall, 103 suspected RVF cases were identified among 71 households in the four communes. The sex ratio was 4.4 (84 males/19 females). The ages ranged from 2 to 71 years (median: 34 years old; IIQ: 21–46).

Three individuals were determined to be positive by qRT-PCR, and ten had anti-RVFV IgM antibodies. Among them, one was positive for both. The RVFV positivity rate was 11.7% (12/103). The delay between the onset of the disease and the blood sample collection ranged from 3 to 33 days (from 1 April to 5 May 2021, corresponding to epi weeks 13 to 17/2021). Each confirmed case came from a different household. Most of the confirmed cases were farmers [91.7% (11/12)] and men (10/12). Their ages ranged from 20 to 48 years (median: 29 years old; IIQ: 24–44). Positive cases of RVFV were identified in three communes (Table 1): one in Andonabe (one IgM+), seven in Anosimparihy (four IgM+, 2 qRT-PCR+, and one IgM+/qRT-PCR+), and four in Antsenavolo (four IgM+). Two cases with hemorrhagic symptoms were hospitalized, and one case died (Table 2).

Eight confirmed cases reported symptoms of RVF (Table 2). All suffered from headaches. The main other reported symptoms were fever (7/8), myalgia (5/8), asthenia (5/8), and abdominal pain (5/8).

None of the confirmed cases reported consuming raw milk, 83.3% (10/12) had been in contact with cattle, 83.3% (10/12) had been in contact with deceased cattle suspected of RVF, and 33.3% (4/12) has been in contact with abortion products. One confirmed human case and at least one concomitant confirmed animal case were identified in three households, implicating two male farmers from Anosimparihy and a female teacher from Andonabe.

## 4. Discussion

Our results suggest a recent circulation of RVF from epidemiological weeks 5 to 18/2021 in the cattle, confirmed by serological testing in four communes (Ambohimiarina II, Anosimparihy, Antsenavolo, and Andonabe) of the Mananjary district in the Vatovavy Fitovinany region. In humans, twelve cases were confirmed by qRT-PCR and/or IgM testing between weeks 13 to 17/2021 in three of the four communes (Antsenavolo, Anosimparihy, and Andonabe) investigated: three were asymptomatic, six had mild symptoms, and three had severe symptoms. The case fatality rate was 8% (1/12).

Our results suggest that viral transmission continued in humans during the investigation. Previous studies have shown that consumption of or contact with infected animals or their products can potentially infect humans [12,13]. Our data show that most confirmed cases were in contact with cattle that were suspected of having or confirmed to have RVF. However, we did not find a statistically significant association, probably due to the small sample size. An entomological survey was conducted in parallel with this survey. The entomological results concerning the possible role of local populations of mosquitoes in virus transmission during this outbreak are presented in a separate paper [14]. While the virus can persist for years in the environment [15,16,17], the importation of RVFV from other countries has also been implicated in previous epidemics in Madagascar and other countries [18,19,20]. Further molecular analyses will be carried out to determine the origin of the strain of RVFV responsible for this epidemic.

In the course of this survey, the DVSSE-R team set up a public education program to explain prevention and control measures, such as sanitary measures and livestock quarantine to prevent the spread of the disease, to CHWs and neighborhood chiefs in the communes investigated.

The clinical symptoms of RVF are not always specific [21]. In Kenya, a significant delay in the detection of suspected and confirmed cases was attributed to the low sensitivity of passive surveillance, which is used for RVF surveillance [22,23]. In Uganda, during the COVID-19 pandemic in 2021, delays in accessing healthcare centers for patients with RVF were reported [24]. In Madagascar, a delay may be attributed to the concurrent occurrence of the RVF outbreak and the COVID-19 wave observed during the first months of 2021 [25,26]. The various non-pharmaceutical measures implemented during the COVID-19 outbreak made travel between or within regions very difficult. Consequently, sending samples and the movement of the investigation team became challenging.

Our results showed an 8-week delay between the time when suspected animal cases were reported and the confirmation of human cases. In Mayotte, human cases were reported during the rainy season in the 2018–2019 period. Further investigations suggested that the virus had been imported from neighboring countries through illegal livestock trading during the dry season and circulated at low levels for several months before being detected [27,28]. The detection of human cases indicates that RVFV has already been circulating for some time [22]. Having confirmed the circulation of RVFV in animals, control measures must therefore be taken to reduce the health burden of RVF in humans.

Madagascar was affected by two RVF epizootics/epidemics in the 1990–1991 and 2008–2009 periods, with a long inter-epizootic period (12 years). Multiple factors have been reported to contribute to the emergence of RVF, such as the presence of susceptible hosts, the presence and proliferation of mosquito vectors, vertical transmission in mosquitoes, and favorable environmental conditions [13,29]. A serological survey carried out in ruminants after the 2008 epidemic confirmed the circulation of RVFV, including in areas where no clinical animal or human disease had been reported [30]. There is therefore an urgent need to strengthen RVF surveillance within the framework of the “One Health” concept, including syndromic and risk-based surveillance [31,32,33]. Entomological surveillance is effective but challenging to implement and maintain in the long term. In any case, vector control should be adapted according to the local specificity of each region. Measures should be implemented to strengthen animal surveillance, which is of both economic and public health interest. To make the implemented actions more effective, information about virus circulation should be shared as soon as the confirmation has been made. Notification upon detection is an essential element for the prevention and control of RVF [34,35]. Actions must be taken in advance of an epidemic and be dictated in a dedicated contingency plan to fight RVF.

Emphasis must be placed on coordinating information sharing between animal and public health stakeholders in order to define prevention and control measures to tackle RVF on national and regional scales.

## Figures and Tables

**Table 1:** Results of molecular and serological screening for RVFV by commune.

	Human			Cattle		
Commune	Samples Collected N	qRT-PCR + n (%)	IgM + n (%)	Samples Collected N	IgM + n (%)	IgG + n (%)
Anosimparihy	45	3 (6.7)	5 (11.1)	16	8 (50.0)	9 (56.3)
Antsenavolo	47		4 (8.5)	16	3 (31.3)	11 (68.8)
Andonabe	9		1 (11.1)	5	2 (40.0)	2 (40.0)
Ambohimiarina II	2			3	1 (33.3)	2 (66.7)
Total	103	3 (2.9)	10 (9.7)	40	16 (40.0)	24 (60.0)

**Table 2:** Characteristics and lab results of confirmed cases.

Date of Onset	Communes	Gender	Age (Year)	Profession	Headache	Fever	Myalgia	Asthenia	Abdominal Pain	Articular Pain	Hemorrhagic Sign	Palmar Pallor	Ocular Symptom	Other	Positive Lab Result	Final Status
01/04	Antsenavolo	M	41	Farmer	Yes			Yes	Yes						IgM	
09/04	Antsenavolo	M	20	Farmer	Yes	Yes									IgM	
14/04	Andonabe	F	26	Teacher	Yes	Yes									IgM	
16/04	Anosimparihy	M	23	Farmer	Yes	Yes	Yes		Yes	Yes	Yes		Yes	Neck pain, anorexia	IgM	
19/04	Anosimparihy	M	46	Farmer	Yes	Yes	Yes	Yes	Yes	Yes				Backpain, chills	IgM	
19/04	Anosimparihy	M	48	Farmer	Yes	Yes	Yes	Yes	Yes				Yes	Diarrhea, chills	qRT-PCR	
26/04	Anosimparihy	F	26	Farmer	Yes	Yes	Yes	Yes	Yes	Yes		Yes			qRT-PCR	
01/05	Anosimparihy	M	33	Farmer	Yes	Yes	Yes	Yes		Yes	Yes	Yes		Jaundice, splenomegaly	qRT-PCR, IgM	Deceased
ND	Antsenavolo	M	26	Farmer											IgM	
ND	Anosimparihy	M	21	Farmer											IgM	
ND	Anosimparihy	M	46	Farmer											IgM	
ND	Antsenavolo	M	42	Farmer											IgM	

ND: No Data; M: Male; F: Female.

## Data Availability

The data that support the findings of this study are available from the corresponding author upon reasonable request.
